# Vitamin C and Microvascular Dysfunction in Systemic Inflammation

**DOI:** 10.3390/antiox6030049

**Published:** 2017-06-29

**Authors:** Karel Tyml

**Affiliations:** 1 Centre for Critical Illness Research, Lawson Health Research Institute, London, ON N6A 5W9, Canada; karel.tyml@lhsc.on.ca; 2 Department of Medical Biophysics, University of Western Ontario, London, ON N6A 5C1, Canada

**Keywords:** sepsis, microvessels, endothelial cells, platelets, electrical coupling, connexins, nitric oxide, P-selectin, coagulation, plasminogen-activator-inhibitor-1

## Abstract

Sepsis, life-threatening organ dysfunction caused by a dysfunctional host response to infection, is associated with high mortality. A promising strategy to improve the outcome is to inject patients intravenously with ascorbate (vitamin C). In animal models of sepsis, this injection improves survival and, among others, the microvascular function. This review examines our recent work addressing ascorbate’s ability to inhibit arteriolar dysfunction and capillary plugging in sepsis. Arteriolar dysfunction includes impaired vasoconstriction/dilation (previously reviewed) and impaired conduction of vasoconstriction/dilation along the arteriole. We showed that ascorbate injected into septic mice prevents impaired conducted vasoconstriction by inhibiting neuronal nitric oxide synthase-derived NO, leading to restored inter-endothelial electrical coupling through connexin 37-containing gap junctions. Hypoxia/reoxygenation (confounding factor in sepsis) also impairs electrical coupling by protein kinase A (PKA)-dependent connexin 40 dephosphorylation; ascorbate restores PKA activation required for this coupling. Both effects of ascorbate could explain its ability to protect against hypotension in sepsis. Capillary plugging in sepsis involves P-selectin mediated platelet-endothelial adhesion and microthrombi formation. Early injection of ascorbate prevents capillary plugging by inhibiting platelet-endothelial adhesion and endothelial surface P-selectin expression. Ascorbate also prevents thrombin-induced platelet aggregation and platelet surface P-selectin expression, thus preventing microthrombi formation. Delayed ascorbate injection reverses capillary plugging and platelet-endothelial adhesion; it also attenuates sepsis-induced drop in platelet count in systemic blood. Thrombin-induced release of plasminogen-activator-inhibitor-1 from platelets (anti-fibrinolytic event in sepsis) is inhibited by ascorbate pH-dependently. Thus, under acidotic conditions in sepsis, ascorbate promotes dissolving of microthrombi in capillaries. We propose that protected/restored arteriolar conduction and capillary bed perfusion by ascorbate contributes to reduced organ injury and improved survival in sepsis.

## 1. Introduction

Local infectious or non-infectious insult can lead to a systemic inflammatory response. Sepsis, life-threatening organ dysfunction caused by a dysfunctional host response to infection [1], can precipitate multiple organ failure and 40% mortality in Intensive Care Units [2]. Sepsis is annually responsible for the loss of more lives than breast, colorectal, pancreatic and prostate cancers combined [3]. The prevalence of septic patients is highest in the elderly (i.e., older than 65 years) where the outcome disproportionately worsens with age [4]. Sepsis involves many pathophysiological processes including increased oxidative stress [5]. The age-aggravated worsening of outcome could be due to increased mitochondrial free radical formation that occurs naturally in aging tissues [6,7].

Among all antioxidants, ascorbate (reduced vitamin C) is considered to be the most effective water-soluble antioxidant [8]. In healthy middle-age humans, plasma ascorbate concentration is 60–80 μM [6,9], but in healthy elderly it drops to ~40 μM [10,11]. Critically, in the septic elderly, plasma ascorbate is clinically considered to be depleted (i.e., ~10 μM) [12,13,14]. Thus, the major defense by the antioxidant ascorbate against sepsis is nearly absent in the elderly.

A promising strategy to improve the outcome of sepsis is to replete ascorbate in patients quickly after the diagnosis of sepsis [15]. Indeed, a recent clinical study including the septic elderly reported markedly improved survival in patients injected intravenously with vitamin C, hydrocortisone and thiamine [16]. This improved survival is consistent with that observed in septic mice injected with ascorbate [15,17,18].

The sepsis-induced inflammatory response leads to dysfunction of many organ systems, including the cardiovascular system where decreased systemic vascular resistance, hypotension, maldistribution of blood flow in the microcirculation, and impaired oxygen utilization occur [19,20]. Using various animal models of sepsis, our laboratory has examined the dysfunction of the microcirculation, and the possible beneficial effects of intravenous injection of ascorbate against this dysfunction. The models, the dysfunction, and the effects of various doses of ascorbate have been reviewed [5,15,21,22]. However, our recent advances in this area extend our understanding of protection by ascorbate against microvascular dysfunction in sepsis. The objective of the present paper is to review these advances.

## 2. Arteriolar Dysfunction in Sepsis

Within the systemic vascular system, arterioles represent the key site along the vascular tree responsible for both the control of blood supply to the tissue and the peripheral vascular resistance [23]. The vascular resistance (and flow control) depend on (i) the degree of arteriolar diameter change elicited by local physiological/pharmacological stimuli impinging on the arteriolar wall, and (ii) the degree of conduction (or spread) of the diameter change along the arteriolar length [24]. A local arteriolar dilation without conduction yielded no increase in blood flow in the microvascular network fed by the stimulated arteriole [25].

We have shown that sepsis (cecal ligation and perforation, CLP) in young mice impairs norepinephrine-induced vasoconstriction in 6–10 µm arterioles in skeletal muscle, and that ascorbate intravenous injection protects against this impairment [26]. Similar protection by ascorbate in the vasculature has been shown for other vasoconstrictors as well as for vasodilators [21,27,28]. The mechanism of protection by ascorbate against impaired vasoconstriction has been reviewed [15]. It involves (i) inhibition of nicotinamide adenine dinucleotide phosphate (NADPH) oxidase and inducible nitric oxide synthase (iNOS) in endothelial cells of the vascular wall and (ii) inhibition of the subsequent refractory vasodilation caused by iNOS-derived NO.

### 2.1. Arteriolar Conducted Response in Vivo

In addition to the arteriolar diameter response, we have also examined the effects of sepsis and ascorbate on arteriolar conduction. The conduction is underpinned by electrical coupling along the arteriolar endothelial layer where connexins (i.e., constituents of inter-cellular gap junctions) are required for this coupling [29]. We used CLP (24 h model of sepsis) and lipopolysaccharide (LPS) in young mice to show that sepsis impairs conducted vasoconstriction in skeletal muscle by tyrosine kinase- and NO-dependent mechanisms [24,30]. We further determined that this impairment is mediated by the neuronal NOS (nNOS)-derived NO production and that the target of NO signaling could be the gap junction protein connexin 37 (Cx37) in the arteriolar wall [31]. Finally, we showed that an intravenous bolus of ascorbate prevented as well as reversed impairment of conducted vasoconstriction at 24 h of sepsis by inhibiting nNOS-derived NO production [32].

These studies indicate that, in addition to iNOS, nNOS is also an important source of vascular NO in our in vivo model of sepsis. Here, nNOS is found in smooth muscle cells and adjacent skeletal muscle cells [33,34]. It is possible that the protective effect of ascorbate against impaired conduction involves the heat shock protein 90 (HSP 90). HSP 90 is up-regulated during sepsis [35] and, when it binds to nNOS, it increases nNOS activity [36]. Because sepsis increases the level of reactive oxygen species (ROS) in skeletal muscle [26], and HSP 90 protein expression increases in response to ROS [37], ascorbate could scavenge ROS, prevent HSP 90 protein up-regulation, inhibit sepsis-induced increased nNOS activity and NO production, and thus prevent the septic impairment of arteriolar conduction [29].

### 2.2. Inter-Endothelial Electrical Coupling In Vitro

In order to gain further mechanistic insights into the impairment of conduction, we have developed an electrophysiological approach to determine inter-endothelial electrical coupling under conditions that mimic sepsis. We used monolayers of cultured microvascular endothelial cells obtained from the mouse skeletal muscle (i.e., the same tissue studied in vivo). Regarding the role of nNOS-derived NO in the impairment, we mimicked sepsis by applying exogenous NO to the monolayer. NO reduced electrical coupling [38]. Using cells from mice where individual vascular connexins were knocked-out, we determined that NO indeed targets Cx37 to reduce coupling [38]. This reduction could be due to the effect of peroxynitrite (i.e., formed after NO reaction with superoxide), or due to a direct effect of NO. Pretreatment of monolayers with ascorbate or with peroxynitrite scavenger did not affect the reduction in coupling, indicating that NO reduces coupling directly, possibly via Cx37 nitrosylation [38]. Thus, ascorbate appears to protect against impaired arteriolar conduction sepsis by affecting arteriolar function indirectly (i.e., reducing nNOS activity and NO production), rather than by affecting the target molecule Cx37.

In addition to addressing the mechanism of impaired conduction during the advanced stage in sepsis involving NO (i.e., 24 h post-CLP), we also used our electrophysiological approach in endothelial cell monolayers to address the mechanism of impaired conduction caused by LPS (i.e., an initiating factor in sepsis). We discovered that LPS reduces inter-endothelial electrical coupling via tyrosine-, ERK1/2-, PKA-, and PKC-dependent signaling that targets Cx40 [39]. This finding was consistent with the LPS-induced tyrosine-dependent impaired conduction observed in arterioles in vivo [24].

Importantly, impaired arteriolar dilatation/constriction and conduction in sepsis results in impaired microvascular blood flow which, in turn, precipitates episodes of micro-regional ischemia/reperfusion (I/R) in the tissue supplied by the arteriole (i.e., evidenced by intermittent capillary blood flow in septic skeletal muscle, [40]). I/R has been shown to aggravate the sepsis-induced inflammatory response [41,42]. Because ascorbate prevents the development of intermittent capillary blood flow in sepsis in vivo [40], we also sought to determine if ascorbate protects against reduction in inter-endothelial electrical coupling in our endothelial cell monolayer model in vitro. Using a hypoxia/reoxygenation (H/R) protocol to mimic I/R, we discovered that (i) H/R reduces inter-endothelial coupling PKA-dependently, also by targeting Cx40, and (ii) ascorbate pretreatment of the monolayer prevents this reduction by scavenging ROS [43]. This scavenging eliminates PKA inhibition by ROS [43]. Significantly, we were able to corroborate the aggravating effect by I/R on sepsis-induced inflammatory response. Concurrent LPS+H/R application to the monolayer synergistically reduced inter-endothelial electrical coupling, PKA- and PKC-dependently [44]. We demonstrated that LPS+H/R initiates tyrosine kinase- and ERK1/2-sensitive signaling that reduces electrical coupling by dephosphorylating PKA-specific serine residues of Cx40 [44]. Our most recent work pinpointed the residues 345–358 of the Cx40 carboxyl terminal tail as possible sites of this dephosphorylation [45].

Taken together, a complex picture emerges for the impaired arteriolar conduction in sepsis. Initially, LPS and the concurrent H/R may reduce inter-endothelial electrical coupling and arteriolar conduction by targeting Cx40, whereas nNOS-mediated NO overproduction in advanced sepsis reduces coupling and conduction by targeting Cx37 instead. The protection by ascorbate against impaired conduction involves (i) inhibition of the H/R component in the initial stage of sepsis (i.e., ascorbate restores PKA activation required for conduction) and, in the advanced stage, (ii) inhibition of nNOS activation and excess NO production.

Protection by ascorbate against both sepsis-induced impairment in arteriolar vasoconstriction and conduction could explain ascorbate’s ability (i) to inhibit hypotension in rat models of sepsis [40,46] and (ii) to markedly reduce duration of vasopressor treatment in septic patients, normally necessitated by falling blood pressure [16].

## 3. Capillary Plugging in Sepsis

Sepsis-induced inflammation leads to activation of the coagulation pathway [47]. We have used the skeletal muscle in rats and mice as a bioassay to examine this aspect of sepsis in terms of capillary bed plugging, a well-known indicator of sepsis involving pro-coagulant responses [5,15]. Capillary plugging involves P-selectin mediated platelet-endothelial adhesion and fibrin deposition in capillaries [48,49]. This plugging, reported in animal and human organs, leads to inadequate oxygenation of the tissue and organ failure [5,15]. Importantly, we and others have shown that intravenous injection of ascorbate protects against sepsis-induced capillary plugging and organ injury [17,40,48,50]. The experimental details and the mechanism of this protection against capillary plugging have been reviewed [5,15]. A key component of this mechanism is endothelial nitric oxide synthase (eNOS) in endothelial cells of the microvascular wall. Since eNOS-derived NO is anti-coagulatory (i.e., it reduces platelet-endothelial adhesion [51]), and since the protection by ascorbate is absent in eNOS^−/−^ mice [50], ascorbate has been proposed to act indirectly via restoring the eNOS function in the microvasculature in sepsis [50].

Ascorbate intravenous injection early in sepsis prevents capillary plugging, whereas delayed ascorbate injection later in sepsis reverses plugging (i.e., restores blood flow in previously plugged capillaries) [40,46,48]. Recently, we have addressed the mechanisms of both the prevention and reversal of plugging.

### 3.1. Ascorbate Prevents Capillary Plugging in Sepsis

A key event in the initiation process of sepsis-induced capillary plugging is platelet adhesion to the capillary wall. Pretreatment of mice with platelet-depleting antibody inhibits this plugging [48]. P-selectin is a key platelet-endothelium adhesion molecule [52]. Pretreatment of mice with P-selectin blocking antibody also inhibits plugging [48]. We have carried out a series of experiments designed to tease out whether platelet surface and/or endothelial surface P-selectin expression are involved in the initiation. Using a platelet-endothelial cell adhesion assay in vitro, we determined that activation of endothelial cells by LPS increased platelet adhesion to the endothelial monolayer (mouse skeletal muscle origin) P-selectin-dependently [49]. Further, LPS increased P-selectin protein expression at the surface of endothelial cells, most likely by promoting exocytosis of P-selectin protein already contained in Weibel-Palade granules beneath the endothelial surface [49,53,54]. Significantly, pretreatment of the monolayer with ascorbate inhibited all platelet adhesion to the monolayer, endothelial P-selectin surface expression, and exocytosis [49]. These in vitro studies demonstrated that ascorbate can inhibit platelet-endothelial adhesion in capillaries in vivo directly, rather than indirectly via hemodynamic effects of ascorbate on capillary blood flow.

We have also used a platelet aggregation assay in vitro, to examine the role of P-selectin at the platelet surface. LPS or plasma from septic mice did not alter P-selectin expression at the platelet surface, or platelet aggregation [55]. However, platelet-activating agents known to be released into the bloodstream during sepsis [thrombin, adenosine diphosphate (ADP), thromboxane A2] did increase P-selectin expression and aggregation. Interestingly, ascorbate inhibited these increases independently of platelet-derived NOS [55]. Thus, ascorbate could reduce aggregation directly, independent of its ability to restore eNOS function within the microvasculature. In the context of plugging of septic capillaries, the inhibition of platelet aggregation directly by ascorbate may not be enough to fully prevent plugging. In addition to this direct effect, NO-derived from non-platelet sources (e.g., endothelial eNOS) may be needed for the full in vivo effect of ascorbate.

Our results suggest a complex mechanism in the initiation of capillary plugging and in the protection by ascorbate against this plugging. An early platelet-endothelial adhesion may be followed/paralleled by the generation of platelet-activating stimuli which, in turn, result in platelet aggregation and buildup of other materials (e.g., fibrin deposits, microthrombi) which eventually plug capillaries [48,55]. So far our data indicate that ascorbate can protect against the initial platelet-endothelial adhesion and the subsequent platelet aggregation in the septic capillary. These data are consistent with the reported anti-coagulatory effect of ascorbate in sepsis [17,18].

### 3.2. Ascorbate Reverses Capillary Plugging

A delayed intravenous injection of ascorbate reverses both the number of plugged capillaries and platelet adhesion/trapping therein (observed in skeletal muscle, in a mouse model of sepsis involving feces injection into peritoneum, FIP) [48]. This reversal is eNOS-dependent [48]. Since platelet trapping in capillaries leads to subsequent reduction in the number of platelets available for detection in systemic blood, the platelet count in systemic blood is a complementary, clinically relevant [56], measure of capillary plugging. To this end, we showed that (i) sepsis at 7 h post-FIP indeed reduces platelet count measured in samples of arterial blood and (ii) ascorbate injection delayed to 6 h post-FIP attenuates this reduction (i.e., previously trapped platelets were released back into the systemic circulation) [48,57].

To address ascorbate’s ability to quickly reverse platelet trapping (i.e., over 1 h period) we examined ascorbate’s ability to dissolve microthrombi in capillaries. This dissolving will permit restarting of blood flow in these microvessels. To this end, we examined the thrombolytic system. Using our FIP model of sepsis in mice, sepsis increased mRNA of both the pro-fibrinolytic urokinase plasminogen activator (u-PA) and the anti-fibrinolytic plasminogen activator inhibitor 1 (PAI-1) in muscle and liver homogenates [57]. Delayed ascorbate did not affect u-PA mRNA in either tissue; it inhibited PAI-1 mRNA in the muscle (i.e., suggesting enhanced fibrinolysis in this tissue) but not in the liver. Since liver PAI-1 is the dominant source of soluble PAI-1 in systemic blood, we further examined PAI-1 enzymatic activity in this tissue. Ascorbate did not affect sepsis-induced increase in PAI-1 activity in the liver [57]. Consistently, delayed ascorbate also did not affect sepsis-induced increase in PAI-1 protein and activity in systemic blood plasma [57,58]. Thus, based on the PAI-1 protein/enzymatic activity data measured in tissue homogenates and systemic blood, our study did not support the hypothesis that ascorbate reverses capillary plugging in sepsis by promoting fibrinolysis.

Local pro- and anti-fibrinolytic events, which occur at the level of the capillary, may not necessarily be assessed by analyzing tissue homogenates or systemic blood. A clear example of this is the observation that sepsis causes hypocoagulability in systemic blood but hypercoagulability in the microcirculation [15]. To address this issue, we used our in vitro models of cultured microvascular endothelial cells (mouse skeletal muscle origin) and platelets isolated from mice (i.e., both cell types are present in the milieu of capillary microthrombi) [59]. Because both cell types can release PAI-1 into the extracellular space [60,61], we asked whether ascorbate affects PAI-1 release from these cells. We used thrombin or LPS to mimic sepsis. In unstimulated endothelial cells and platelets, PAI-1 was released into the extracellular space and this release was unaffected by ascorbate pretreatment. Thrombin or LPS did not alter PAI-1 release from endothelial cells. However, thrombin, but not LPS, increased PAI-1 release from platelets. Ascorbate inhibited this release pH-dependently [59].

Thus, under acidotic conditions prevalent in sepsis, our in vitro studies suggest that, together with the inhibition by ascorbate of thrombin-induced platelet aggregation discussed above, inhibition by ascorbate of thrombin-induced PAI-1 release from platelets would yield a pro-fibrinolytic effect leading to dissolving of microthrombi in septic capillaries.

### 3.3. A Multifaceted Mechanism of Capillary Plugging

Our work has focused mainly on the role of endothelial cells and platelets in capillary plugging observed by intravital microscopy in the septic skeletal muscle. Clearly, there are other cell types which could contribute to the formation of microvascular microthrombi. These include red blood cells which become stiff in sepsis and thus may obstruct the capillary lumen [15,62]. Additionally, activated leukocytes, including neutrophils, can adhere to the capillary/venular endothelium and thus increase the hemodynamic resistance to blood flow. The number of adhering leukocytes was negligible in capillaries and venules in the skeletal muscle of septic mice; these cells thus could not account for the capillary plugging, or be involved in the inhibitory effect of ascorbate against plugging in this tissue [48,62]. However, the presence of neutrophils in immunogenic organs such as the lung and liver and their abundance there during sepsis [18,59] would undoubtedly contribute to capillary plugging therein. Recent reports of neutrophil extracellular traps (NETs) contributing to platelet aggregation or leukocyte-platelet aggregation [63], and to microthrombi formation [64], underscore the involvement of neutrophils in capillary plugging. Importantly, ascorbate has been shown to reduce the lung NETs formation in septic mice [65].

Sepsis leads to endothelial barrier dysfunction, involving increased permeability in the microvasculature and increased extravasation of plasma proteins and fluid (reviewed by [15]). This extravasation could form tissue edema, compress the capillary lumen, and thus also contribute to capillary plugging in sepsis. Ascorbate can inhibit this dysfunction by inhibiting NADPH oxidase expression and activity in endothelial cells, by attenuating protein phosphatase 2 (PP2A) activation, and subsequently restoring the phosphorylation and distribution of the tight junction protein occludin in endothelial cells (mechanism reviewed by [15]).

## 4. Unresolved Issues and Future Directions in Experimental Studies of Systemic Inflammation

Most experimental studies of sepsis have used young animals, but the majority of septic patients are elderly. In septic mice, it has been shown that the levels of plasma inflammatory cytokines, antioxidant defense, and mortality markedly worsen in aged when compared to young mice [66,67,68]. Thus, studies in young animals may have a limited impact on our understanding and development of therapeutic strategies to treat sepsis in the elderly.

To our knowledge, there are no reports addressing the effect of ascorbate on the outcome of sepsis in aged mice. Relevant to this unresolved issue is a recent study using Gulo^−/−^ mice [69]. These mice are deficient in endogenous vitamin C production and thus require the vitamin supplementation in diet. In Gulo^−/−^ mice without supplementation (i.e., mimicking the nearly-depleted plasma ascorbate status in the septic elderly), sepsis resulted in exacerbated mortality and organ injury when compared to both Gulo^−/−^ mice with ascorbate supplementation and Gulo^−/−^ mice injected with ascorbate after the onset of sepsis [69]. The study suggests that ascorbate repletion would be critical when treating the septic elderly.

Because of the increased incidence of obesity in the present general population, another unresolved issue may be the effect of ascorbate on the outcome of sepsis in obese animals or in animals with other co-morbidities. Similar to the effect of aging, obesity in mice also worsens the inflammatory response to sepsis [70]. However, in a clinical study, sepsis in obese patients [71] did not worsen the outcome as predicted by this animal study, underscoring the complexity of human sepsis. To our knowledge, there are no reports addressing the effect of ascorbate on the outcome of sepsis in obese animals.

The non-infectious insult I/R also leads to a systemic inflammatory response, including lung injury [72], impaired arteriolar conduction [43] and capillary plugging [73]. Ascorbate has been shown to attenuate the I/R-induced injury [72] and H/R-induced impairment of inter-endothelial electrical coupling [43]. A key feature of the H/R-induced coupling impairment is H/R-stimulated increase in ROS production in endothelial cells [43,74]. Intriguingly, the stimulated ROS increase is Cx40-dependent, possibly involving a cross-talk between Cx40 and NADPH oxidase [74]. Thus, Cx40 may not function only as a structural protein in intercellular gap junctions [29,75], but also as a signaling molecule responsible for the H/R-stimulated ROS increase in endothelial cells. Given the reported aggravation by I/R in sepsis-induced inflammatory response [41,42], and the critical roles of NAPDH oxidase in impaired arteriolar vasoconstriction in sepsis and of Cx40 in impaired electrical coupling in endothelial cells exposed to LPS, H/R or LPS+H/R, the possible signaling function of Cx40 warrants further investigation.

## 5. Conclusions

Despite numerous animal studies and clinical trials, the mortality in sepsis remains unacceptably high. A promising strategy to improve the outcome of sepsis is to intravenously inject patients with ascorbate (vitamin C) to quickly restore its levels in blood plasma and tissues. We have shown that intravenous injection of ascorbate improves the microvascular function in septic rats and mice. These improvements include the arteriolar responsiveness to vasoactive stimuli and capillary bed perfusion.

In particular, our recent work demonstrated that ascorbate inhibits the sepsis-induced impairment of arteriolar conducted vasoconstriction by inhibiting nNOS-derived NO production and ROS production, to restore the inter-endothelial cell electrical coupling and gap junction function. These effects contribute to ascorbate’s ability to protect against hypotension in sepsis. Further, we demonstrated that ascorbate inhibits capillary plugging in sepsis by inhibiting platelet-endothelial adhesion and platelet aggregation mediated by P-selectin, and by promoting the dissolution of microthrombi in capillaries. These effects contribute to ascorbate’s ability to protect against tissue injury and to improve survival in sepsis.
